# Engaging the private sector to increase access to HIV services and increase sustainability of the HIV response

**DOI:** 10.1186/s12913-025-12530-1

**Published:** 2025-05-28

**Authors:** Hannah Marqusee, Ryan Schowen, Patrick Amanzi, Susanna Baker, Peter Egena, Mai Hijazi, Isa Iyortim, Abimbola Kola-Jebutu, A. K. Nandakumar, Babatunji Odelola, Gene Peuse, Carolina Piña, Jemeh Pius

**Affiliations:** 1 Office of Public Health and Education, USAID Cambodia, U.S. Agency for International Development, Phnom Penh, Cambodia; 2 Office of HIV/AIDS, Bureau for Global Health, U.S. Agency for International Development, Washington D.C., USA; 3 Health Office, USAID Zambia, U.S. Agency for International Development, Lusaka, Zambia; 4 Office of HIV/AIDS and Tuberculosis, USAID Nigeria, U.S. Agency for International Development, Abuja, Nigeria; 5 Health Office, USAID Botswana, U.S. Agency for International Development, Gaborone, Botswana; 6 Office of U.S. Global AIDS Coordinator, Washington D.C., USA; 7 Health Office, USAID Tanzania, U.S. Agency for International Development, Dar es Salaam, Tanzania; 8 Health Office, USAID Dominican Republic, U.S. Agency for International Development, Santo Domingo, Dominican Republic

**Keywords:** HIV/AIDS, Private sector engagement, Service delivery, ART, Blended finance

## Abstract

**Background:**

Public health facilities often manage high volumes of HIV clients and face challenges with long wait times, poor client satisfaction, and low retention in care. The private sector is a major provider of health services in many countries but is a relatively untapped resource for reducing congestion in high-volume public HIV facilities and increasing access to HIV services, particularly for clients who already use the private sector as their first point of care. Private banks, corporations, and clients themselves can also be a resource for generating additional financing for HIV and reducing reliance on donor funding. Through the President’s Emergency Plan for AIDS Relief’s Sustainable Financing Initiative (SFI), USAID engaged the private sector to expand access to HIV care and mobilize private financing for HIV in low- and middle-income countries.

**Methods:**

SFI worked in 11 countries and two regional programs on private sector activities, including technical assistance to financial institutions and private providers on lending to the health sector, facilitating the growth of social enterprise models, enabling private providers to offer additional HIV services, and generating and communicating evidence to host governments to strengthen the policy and regulatory environment for private HIV service delivery. SFI’s impact was estimated through a financial return on investment.

**Results:**

Over 48,000 clients accessed HIV services in the private sector across three countries, and $6.3 million was mobilized in private loans to the health sector in Tanzania. Additional successes in policy, evidence generation, innovative financing, and service delivery were demonstrated globally.

**Lessons learned and recommendations:**

SFI demonstrated the feasibility of delivering high-quality HIV services through the private sector and the high demand for those services; the need for evaluation techniques that incorporate patient experience in addition to program costs, benefits, and clinical outcomes; and the need for long-term strategic donor and government coordination around private sector engagement.

**Conclusions:**

Engaging the private sector in HIV can reduce the burden on public health facilities, provide greater opportunities for access to care, and increase domestic financing for HIV without putting clients into financial hardship.

**Supplementary Information:**

The online version contains supplementary material available at 10.1186/s12913-025-12530-1.

## Background

Meeting UNAIDS’ 95-95−95 goals (identifying 95% of people living with HIV, putting 95% of those on treatment, and ensuring 95% of those on treatment are virally suppressed) by 2025 will require strategies that improve client-centered care, reduce barriers to retention in care, and ensure adequate and sustainable financing for HIV programs. While fourteen countries were able to reach the 90-90−90 targets for 2020, failure to reach this target globally has highlighted deeply unequal success [1]. Reaching the populations left behind will require the use of innovative client-centered approaches, including engaging the private sector to increase convenience, quality, and flexibility of care.

Evidence indicates that inconvenient hours, long wait times, long distances to reach facilities, and stigma are major barriers to antiretroviral therapy (ART) adherence [2]. Partnering with private pharmacies and drug shops, which are often closer to where clients live and work, can eliminate known barriers to adherence and increase patient satisfaction.

The COVID-19 pandemic has also increased urgency to shift services closer to patients and out of overburdened facilities. Throughout 2020 and into 2021, multiple countries faced lockdowns and transportation stoppages that made it difficult to travel to facilities for care [3]. Health care workers have faced increased demands as HIV treatment facilities are often on the front lines of the COVID-19 response. Due to fear of contracting the virus and the increased risk of people living with HIV (PLHIV) to severe COVID-19 [4], patients are less likely to access in-person HIV services. Decentralizing antiretroviral (ARV) dispensing out of facilities and increasing the quantity of medication dispensed to patients through multi-month dispensing are two key strategies used to increase access to medication. Private pharmacies, which are often located in communities, offer a potential solution for patients to access medication without traveling to crowded public facilities.

Most countries with high burdens of HIV have mixed health systems in which the private sector - both commercial and nonprofit - plays a major role in health care delivery [5]. In contrast, government-run public health systems, with support from the President’s Emergency Plan for AIDS Relief (PEPFAR) and other donors, are the dominant providers of HIV services in low- and middle-income countries (LMICs). Use of the private sector for HIV services varies greatly by country, though it is strongly correlated with the use of the private sector for other health services. An analysis of Demographic and Health Surveys in six high-prevalence HIV countries in Africa found that on average, 18% of women and 22% of men who received an HIV test did so in the private sector, similar to rates for STI testing, but much lower than the use of private sector for condoms and family planning [6]. Private providers, including pharmacies, drug shops, clinics, and hospitals, represent a ready supply of infrastructure and skilled health workers that can be tapped into to expand the supply of HIV care.

PEPFAR is the largest international donor for HIV, contributing over $85 billion cumulatively since 2004. After reaching peak levels in 2010, PEPFAR funding has remained relatively flat, and this trend is expected to continue in the future [7]. Host country governments increased funding for HIV by 50% from 2010 to 2017; however, there was still an estimated funding gap of about $7 billion to achieve HIV epidemic control by the end of 2020 [8]. At the same time, the global recession caused by COVID-19 has resulted in decreased fiscal space, declining Gross Domestic Product (GDP) growth, and high government debt levels that will make it even more difficult for countries to make up the shortfall needed to control the epidemic.

Host governments and international donors will be unable to fund HIV epidemic control without greater engagement with the private sector to mitigate market distortions. An analysis of four countries in Africa found that the large influx of donor funding in the early years of the PEPFAR response was associated with a decline in corporate contributions for HIV, a decline in private corporations and insurance agents making decisions about HIV financing, and a decline in funding to informal private providers. This indicates that donor funding likely crowded out the private sector’s contributions to the HIV response [9]. Meanwhile, private lending outside of health, from both foreign and domestic sources, has been on the rise as the primary source of development finance. In the past decade, Development Finance Institutions have seen year-after-year increases in private finance mobilization, growing from $15.3 billion in 2012 to $48.4 billion in 2018 [10]. Financing HIV epidemic control over the long term will require donors and governments to engage with the private sector to mobilize additional resources and reduce market distortions.

Household contributions are also a viable source of financing for HIV services. Evidence indicates a substantial willingness to pay for HIV services among PLHIV, particularly when medication is offered for free, and patients are only charged a small dispensing fee [11–15]. HIV prevalence is also highest among urban, middle, and upper wealth quintile populations, who are most likely to use the private sector for other health care services and are more likely to be willing and able to pay for that care [5, 16]. As many PEPFAR countries grow economically and people become wealthier, willingness to pay for high-quality private sector care will only grow. Long-term financial sustainability may require taking advantage of this trend in consumer preference to increase household financing of HIV services.

Market segmentation, which identifies the diverse preferences of key demographic groups, can help tailor programs to different demographic groups to ensure that subsidies are well targeted to vulnerable populations and that all clients can get affordable care at a location of their choosing, whether in the public or private sector. Many patients also cycle between the public and private sectors throughout their lives. Market segmentation supports access to quality-assured and cost-effective commodities and differentiated services tailored to patient needs and preferences and may therefore promote optimal health outcomes in the long term [17–21].

This paper focuses on efforts to engage the private sector for HIV under the Sustainable Financing Initiative (SFI), which ran from 2015 to 2021 as a $48 million PEPFAR-funded, USAID-led initiative dedicated to mobilizing domestic resources for the HIV response in LMICs [22]. SFI was organized around three programmatic pillars: engaging the private sector, improving public financial management, and ensuring financial protection of PLHIV [23, 24]. Across these three pillars, SFI focused on increasing the efficiency of HIV investments, ensuring sustainable funding for HIV commodities, and generating data and evidence for policy and financing reforms. SFI operated in sixteen countries and two regional programs. This paper outlines the approach and summarizes key results and lessons learned from SFI’s implementation of private sector engagement (PSE) activities, which were conducted in eleven countries and two regional programs and offers recommendations for the next generation of private sector engagement for HIV programs.

## Methods

USAID defines PSE as “a strategic approach to planning and programming through which USAID consults, strategizes, aligns, collaborates, and implements with the private sector for greater scale, sustainability, and effectiveness of development or humanitarian outcomes” [25]. As an agency, USAID’s approach to PSE focuses on partnering with the private sector to solve development challenges more sustainably, with an emphasis on market-based approaches. SFI’s PSE approach focused on market-based or commercial approaches and broader partnerships with the private health sector to expand access to free or highly subsidized HIV services. SFI engaged a broad array of private-sector actors, including for-profit and nonprofit organizations involved in any aspect of the health system, such as service providers (e.g., pharmacies, community-based organizations, or for-profit clinics), banks, private employers, and supply chain actors. Priority activities included service delivery and financing activities. Countries were selected to participate in SFI based on the presence of an active private sector and interest from government counterparts, USAID mission staff, private stakeholders, and clients. Activities were planned and implemented in alignment with routine PEPFAR Country Operational Plan (COP) processes, an annual strategic planning process in which country-level budgets and targets are approved for the upcoming year. SFI was designed to fund innovative or untested ideas that would not have been approved through COPs. After proof of concept, the goal was to continue or replicate successful activities through COP funding.

Data were collected and analyzed through routine monitoring approaches and systems using PEPFAR’s standard indicators and custom indicators collected by implementing partners. Standard indicators include HIV clinical measures related to HIV prevention, testing and counseling, initiation and retention on ART, and viral load coverage and suppression. Data on financial investment and returns were collected and analyzed through specialized analyses of donor funding levels, implementing partner budgets and expenditures, and implementing partner work plans and reports.

In the Results section, we have reported on the activity designs, clinical outcomes, and financial outcomes of a selection of private service delivery and financing activities that are representative of the private sector work of SFI.

### Private service delivery

SFI’s private service delivery activities were divided into three typologies. In the first, private providers were used as a channel to distribute donor or government-funded commodities and services at no cost to patients. In these fully subsidized models, private providers were incentivized to participate by donors. In the Results section, we describe one such activity in Zambia. In the second typology, care was fully commercial (i.e., privately delivered and financed), where clients voluntarily purchased HIV products and services as part of a sustainable marketplace, as occurred in Nigeria. In the third typology, care was privately delivered, and most costs (usually medication) were paid by donors or governments, and clients paid a small, voluntary service fee to make the program more sustainable. Examples of these blended models from Tanzania and Nigeria are described further. For programs where patients paid for services or commodities, analyses were first conducted to ensure that there was willingness and ability to pay these fees, that participation was fully voluntary, and that free alternatives existed in the public sector. Activities in support of private service delivery included data collection and analysis; policy and strategy development; stakeholder engagement; and capacity building to providers. A full list of all SFI activities is included in Additional file 1.

### Private financing

Financing activities sought to mobilize resources from private companies, banks, and households through two strategies. First, blended finance approaches used U.S. government-backed loan guarantees and capacity building to incentivize greater private lending to the health sector, which was implemented in Tanzania using a Development Credit Authority guarantee. Second, SFI provided capacity building to nongovernmental organizations (NGOs) to launch revenue-generating social enterprises, which was implemented in the Dominican Republic.

### Outcome metrics

SFI placed a unique emphasis on quantitative outcome reporting and linking health system-wide gains back to HIV-specific gains. Quantitative outcomes were measured in terms of both clinical and financial metrics. Clinical outcomes focused on the number of clients accessing care, and when data were available, other outcomes, such as the percent of cases testing positive linked to treatment and the percent of clients retained in treatment. Clinical data were provided by implementing partners who routinely collect and report these data as standard indicators on a quarterly basis through PEPFAR data systems.

Financial returns were calculated as a return on investment (ROI), which is the net financial benefit of an intervention divided by the initial investment to implement that intervention. SFI activity budgets, inclusive of program management costs, were used to represent the investment amount and were unadjusted to present dollars for simplicity. The financial benefit of service delivery activities was calculated from the perspective of host governments as the cost of care avoided by the public sector as a result of moving patients to the private sector, using available literature on the unit cost of care in the public sector. This assumed that reducing patient load in public facilities frees up staff time, supplies, and other resources that can be used for other purposes. Financial benefits were calculated as the total number of services (e.g., self-test kits, ARV refills, or viral load tests) delivered in the private sector multiplied by the estimated variable unit cost of service delivery in the public sector. The variable unit cost was taken from published literature on the variable unit cost of a service (e.g., HIV testing, ARV refill visit, or viral load testing). User fees and other opportunity costs incurred by the patient were not included in this analysis but may account for additional benefit. More details on the source data behind these calculations are available upon request to the authors.

Financial returns for financing activities were calculated as the amount contributed by private banks, companies, or foundations in loans or grants as a result of the intervention. An ROI was not calculated for activities that lacked quantitative outcomes or strictly focused on policy development or data analysis. ROI for both service delivery and financing activities also did not factor in cost-share by host governments or other USAID funding streams outside of SFI, apart from the Zambia service delivery activity, where other USAID co-funding was a significant portion of the total funding.

## Results

Additional file 1, attached, includes a description of PSE activities for all countries, major results, and available financial ROI over the life of SFI. More detailed results of activities highlighted in the previous section are included here.

### Service delivery results

Over 48,000 clients accessed testing, treatment, or viral load testing services across Zambia, Nigeria, and Tanzania. Clinical outcomes were as strong, or better, than outcomes in the public sector across these three country programs.

In Zambia’s Ndola district, a high-prevalence area with approximately 48,000 clients on ART, SFI supported the expansion of a Central Dispensing Unit (CDU), which centralized the packing, labeling, and distribution of publicly- funded antiretroviral medication (ARVs) to a network of decentralized pickup points, including private pharmacies, electronic lockers, and other community-based locations. In this program, clinically stable clients at any of seven public facilities in Ndola district were able to access six-month refills of medication from decentralized pickup points at no cost. PEPFAR-funded partners managed logistics of medication to dispensing points, and private pickup points were compensated by PEPFAR to incentivize participation in the program [16]. The CDU streamlined the distribution of commodities by directly servicing last mile pickup points, bypassing higher tier health facilities.

Sixteen thousand, six hundred sixty-five clients enrolled in the CDU program, representing over half of those eligible in participating health facilities, and 97% of CDU clients were retained after 36 months [26].

In Nigeria, SFI supported the creation of a network of private community pharmacies. Each private pharmacy was paired with a public “hub” facility to serve as satellite dispensing points for publicly funded ARVs. In this model, clinically stable clients who chose to refill their ARVs at a private pharmacy (generally for three months of medication) paid a small dispensing fee of 500-1,000 Naira ($1.22–2.43 USD) to compensate the private pharmacies for their time. These consultation fees increased the program’s sustainability by reducing the need for donor funds to incentivize private providers to participate in the model. Pharmacists were responsible for collecting ARVs from the hub facilities, distributing them to clients, counseling, and reporting [27]. Among clients accessing ARV refills from private pharmacies in Nigeria, 97% were retained on care after 12 months, compared to 74% in the public sector. They also experienced shorter average wait times of 30 min compared to 2.3 h in the public sector. The success of this program encouraged USAID/Nigeria to replicate it in additional districts and for other USAID missions to adopt it as a model.

Nigeria also piloted market-based approaches where SFI supported private outlets to develop commercial HIV services. This included a partnership with two private labs to help them lease PCR testing equipment for HIV viral load testing and then sell viral load testing services to clients at market rates. A second market-based activity negotiated with local HIV self-testing (HIVST) distributors to enable private pharmacies to purchase HIVST kits at reduced rates and retail them to interested clients.

Uptake of the viral load testing and HIVST activities in Nigeria was limited due to competition from lower-priced alternatives elsewhere. As a result, these activities were not continued or replicated.

In Tanzania, SFI supported private nurse and midwife-run health facilities, which previously had offered HIV testing and PMTCT services to pregnant women and adolescents, to offer HIV treatment services for a wider population, including men. Medication was offered for free, and patients were charged a standard service fee, ranging from 2,000 TSH (approx. $0.90) to 5,000 TSH (approx. $2.25), depending on the service. SFI supported a demonstration project with one private facility through stakeholder engagement; provider capacity building on HIV treatment, referrals, and reporting; supportive supervision and ongoing mentoring for quality improvement; and establishment of a referral network for complex cases. Following the success of the demonstration project, SFI supported the creation of standard operating procedures to be used to replicate the model in other private facilities throughout the country. 7,412 clients at the nurse and midwife-managed clinics received HIV testing services, and 61 clients (100% of those who tested positive) were linked to care.

A second market-based activity in rural Tanzania piloted community-based distribution of ARVs to clients willing and able to pay a delivery fee of 10,000 TZS (about $4 USD). Clinically stable adult clients at two facilities were given the option to receive two-month refills of ARVs drugs in the community; this revenue was designed to cover transport and staff time for the program and would be reinvested in expansion of other community-based services. 200 clients received ARV refills during the first month of the program and 148 in the second month, after which the pilot was put on hold due to challenges with fee collection and requests from clients for additional services, such as treatment for opportunistic infections, viral load testing, and ARV refills for family members who did not meet the eligibility criteria of being clinically stable.

The financial ROI from service delivery activities was limited, with minor or negative ROI reported across several activities due to the large upfront investment required for health systems activities and the short time horizon of the analysis. Zambia, in particular, incurred high capital costs and lower than anticipated uptake. A program evaluation found that while the CDU had enrolled over half of eligible clients in seven partner facilities, this represented far less than its projected capacity, resulting in high per-patient costs. Nigeria’s private lab strengthening activity also generated negative ROI and lower than anticipated uptake due to the high cost of viral load testing and low willingness to pay. However, Nigeria’s private pharmacy program generated a positive ROI of 45% over a three-year period. This figure only reflects an estimate for cost savings to the government and does not factor in contributions from clients in the form of dispensing fees. The Tanzania private nurse-led clinic activity generated negative ROI, likely because of the short time horizon of analysis. Finally, ROI from the Tanzania community-based ART distribution program was not available. Lessons learned and limitations of ROI as a metric for health systems activities are discussed further in the lessons learned section.

In addition to their quantitative outcomes, many of these activities had positive results in terms of patient experience that can only be captured qualitatively. In Nigeria, clients accessing ART from private pharmacies expressed high satisfaction. One interviewed client stated, “I like the program of community pharmacy. It has more privacy. It saves time. I want to continue.” In Tanzania, the community-based ARV distribution pilot demonstrated high demand for community-based HIV services, with over twice the registered clients requesting ARV refills on the first scheduled pickup day. However, this pilot was put on hold in part because many of these clients were not clinically eligible to participate, were returning to care after interruptions in treatment, or needed comprehensive HIV services, such as viral load testing and management of opportunistic infections, which were not available through the pilot.

Cost sharing also improved program sustainability. Following a PEPFAR transition in 2019 that caused SFI’s implementing partner in Nigeria to transition out of Rivers State, the program continued to operate without SFI involvement through the existing partnership between the public facility and private pharmacy network. The Tanzania nurse and midwife-led clinic activity was also highly sustainable; following SFI’s demonstration project, this model was scaled to an additional 69 facilities with additional USAID funding. While it is estimated that about one-third of trained facilities no longer offer the comprehensive set of services due to turnover in trained staff, the model continues to operate for most facilities that received training and commodity support.

### Financing results

In Tanzania, USAID had an existing Development Credit Authority (DCA) agreement with CRDB bank to guarantee private loans of up to $2.8 million to the health sector that had not been used for two years. SFI provided technical assistance to private health facility applicants and responded to CRDB clarification questions, with the result that the DCA was fully utilized within two years. CRDB publicly stated that the private health sector would be a priority investment area for the bank and sought a follow-up DCA. In April 2021, an $8.8 million DCA was established to cement CRDBD’s commitment to financing the private health sector.

In contrast to service delivery activities, the DCA-backed financing activity generated $6.3 M in private loans to the health sector and an ROI of 1,468% (or a total $16 for every $1 invested by USAID) due to the relatively low implementation cost to USAID. This work also demonstrated the feasibility of lending to the health sector without a credit guarantee. Despite the availability of the DCA, only $1.8 million of the loans were issued under the DCA credit guarantee. The technical assistance provided by SFI incentivized CRDB bank to issue the remainder, $4.5 M in loans, without a credit guarantee. Many of these funds were used by health providers to purchase equipment to expand their HIV testing capacity. Among 17 of the loan recipients, there was a 35% increase in HIV tests following the loans [28].

In the Dominican Republic (DR), PEPFAR has historically funded several NGO providers to deliver HIV services at no cost to clients. To ensure the sustainability of these NGOs as PEPFAR funding declines, SFI supported them to develop business plans aimed at increasing their operating efficiency, generating new revenue sources to cross-subsidize HIV services, and improving their marketing and outreach to PLHIV.

As a result, one of these organizations opened a new dermatology center. Under this plan, additional revenue from the new dermatology services would help subsidize routine HIV services. Uptake of dermatology services was slow during the initial years of the COVID-19 pandemic. However, this organization is now in the process of transferring their services to a new location where they anticipate demand will be higher [29]. Financial ROI for this activity was not available at the time of publication.

#### Lessons learned and recommendations

The SFI’s PSE work generated multiple lessons learned and recommendations, including the potential to scale quality HIV services in the private sector to improve access to care and reduce the burden on the public health system; the need to use additional outcome metrics beyond ROI; the necessity of government ownership for policy reform; the need for strategic donor coordination to expand private sector engagement; and opportunities for innovation to transform healthcare.

### There is high demand for quality HIV services in the private sector and interest in integrating HIV with other health needs

SFI’s service delivery work demonstrated the high demand for HIV services in the private sector and, in many cases, high willingness to pay for this care. SFI-funded surveys and focus groups indicated that patients were interested in receiving ARVs in private pharmacies and clinics. This ranged by country for the four countries that conducted client needs assessments, with 12% of clients in Cameroon preferring private pharmacy models compared to 61% in Botswana [30, 31]. More detail on the activities conducted in Cameroon and Botswana can be found in Additional file 1.

SFI’s private service delivery programs demonstrated high quality of care and high patient satisfaction. These results were used as justification to scale and replicate these approaches in additional regions and countries. As of 2022, USAID was implementing ARV refill programs through the private sector in at least 23 countries. In Mozambique, which is adopting a private pharmacy model like the SFI-supported model in Nigeria, an interviewed client explained its perceived advantages:

*“Now I have to ask my boss for time off from work to go to the hospital, and it takes all day to be seen, and my boss doesn’t like it. When somebody asks the boss [for time off] sometimes it takes two days to authorize and I might be without any medicine left. To pick up at the private pharmacy would be good because it’s close and I wouldn’t have to ask my boss anymore because service isn’t delayed there”* [32].

Current private sector models could be strengthened by integrating ARV distribution with other HIV services (e.g., viral load testing and treatment of opportunistic infections) and chronic care services (e.g., tuberculosis, diabetes, and heart disease) and creating eligibility criteria that allow families with multiple members living with HIV to coordinate their care together. Integrating chronic medicines and care coordination into these models will likely increase their long-term sustainability. Other country models, such as the South Africa Central Chronic Medicine Dispensing and Distribution (CCMDD) program, demonstrate that it is feasible and cost-effective to integrate other chronic medications into ARV distribution programs through private pharmacies [33].

### Consider alternative metrics of success in addition to ROI, which does not fully capture the impact of service delivery work

As shown in Additional file 1, many service delivery activities experienced small or negative ROI over the period of analysis. New service delivery models often have high upfront costs due to the need for extensive policy, planning, stakeholder engagement, and capacity building, as well as the occasional need for capital investments, as in the case of the Zambia CDU. The social enterprise models started in the DR are also designed to generate revenue far into the future. Over a longer time horizon, it is likely that many of these activities would see growing ROI along with increased client uptake. In particular, market-based approaches in which clients, rather than donors, pay dispensing fees are much more likely to recoup upfront costs quickly. Future efforts to calculate ROI of health systems activities should consider longer time horizons of analysis or projecting long-term effects into the future.

The ROI methodology employed here did not account for qualitative benefits or economic benefits to patients, which may be significant. Economic evaluations of differentiated care models, including those that use private pharmacies for ARV distribution, show that these models are often cost saving to clients due to reductions in clinic visit times and travel times, but occasionally represent additional costs to providers, donors, and governments [34, 35]. For example, an SFI-funded study found that Nigerian patients could save between $22–52 annually by switching to differentiated care models that reduce the number of annual visits required. This would offset out-of-pocket spending on consultation fees (of approximately $5–10 per year) at private pharmacy models [36].

Finally, it is methodologically challenging to report the ROI of public health activities accurately. For activities that were co-funded with other financing streams, ROI may be overestimated due to the lack of accounting for investments outside of SFI that contributed to program success, including other USAID funding streams and direct or in-kind support from host governments. To address this limitation, ROI for the Zambia intervention was calculated including other USAID co-funding, which was substantial, but data on co-funding from other programs was not available. For financing activities, it can also be difficult to directly attribute financial returns to the USAID investment. These efforts by SFI should be seen as a significant contribution to the results, rather than a direct and exclusive attribution. Focusing primarily on ROI as a key measure of success (or failure) of health systems activities may give short shrift to the importance of policy reform and stakeholder engagement that are inherently difficult to quantify.

Given the limitations with ROI, additional analysis is needed to identify the most cost-effective models of private sector care. Where there is evidence of willingness and ability to pay for services, private sector models that include a cost-sharing component with clients (such as service fees or pre-payment of insurance premiums) may be the most sustainable without ongoing donor support, provided they are voluntary, ensure financial risk protection for PLHIV, and align with domestic universal health coverage and health purchasing policies and priorities [37–39]. Future PSE programs should consider using metrics that demonstrate the financial sustainability of service delivery models (e.g. dollars generated or break-even status) or the replicability/scalability of these models (number of clinics adopting a new model or number of clients accessing services at new locations), as was demonstrated in both the Tanzania nurse-led clinic model and the Nigeria private pharmacy model.

### Government ownership sets programs up for success through enabling policies and strategic leadership

Much of the success of private sector activities is enabled by political will and a supportive policy environment. In Nigeria, extensive engagement with state and federal government and private provider associations contributed to a sense of local ownership and led to successful implementation of the ARV distribution programs. These programs also demonstrated success by piloting innovations at the state level and using those results to advocate for national expansion. SFI’s private pharmacy program began in Lagos and Rivers states before scaling to two additional states (Akwa Ibom and Cross River) after one year and further states after the end of SFI. In contrast, SFI’s efforts to facilitate private pharmacies’ access to HIVST at reduced rates in Nigeria were hampered by a lack of donor and government coordination. After two years of engagement with private pharmacies and HIVST distributors, USAID worked with the government to re-engage a government working group on HIVST which is now dedicated to setting a private sector pricing strategy.

### Donors need to develop and implement long-term coordinated strategies for private sector engagement

SFI’s efforts to increase the sustainability of HIV programs by growing commercial markets and social enterprise models were occasionally undercut by a lack of donor coordination. In the DR, social enterprise activities were designed at a time when PEPFAR signaled that funding to NGOs was likely to decrease. Instead, funding increased in subsequent years, undercutting the perceived need for sustainable models and disincentivizing NGOs from generating revenue.

Several countries also saw lower uptake of commercial services because of competition from fully donor-funded services of comparable quality. For example in Nigeria, ready access to PEPFAR-funded viral load testing suppressed demand and willingness to pay for this service at private labs, which in turn resulted in low volume and a high unit cost. Similarly, efforts to grow the commercial market for HIVST in private pharmacies were undercut by the provision of subsidized HIVST by USAID and other donors at many of the same pharmacies. During SFI’s implementation of community-based ART for a fee, Tanzania had also begun a fully subsidized program of comprehensive care integrated with viral load sample collection, tuberculosis, and family planning in neighboring districts, which suppressed willingness to pay a fee for the SFI-supported program. In these cases, coordination across different donors and long-term strategic engagement could have resulted in better targeting of subsidies so as not to crowd out market-based approaches. SFI’s experience shows that PSE is most successful when donors, implementing partners, host governments, and the private sector are engaged early and often and commit to transparency and coordinated strategic planning.

### Expand innovation with a focus on blended finance and digital technology

SFI’s use of credit guarantees to encourage greater lending to the private health sector was successful and generated a large ROI. However, use of credit guarantees by USAID for HIV has been rare to date. Tanzania’s success in this area may be explained by the existence of a champion at CRDB bank who was personally committed to helping health clinics apply for loans. By partnering with the U.S. International Development Finance Corporation, which uses multiple debt and equity tools to finance the health sector and has field-based staff who are dedicated to identifying and developing financing deals, USAID and PEPFAR can expand credit guarantees and other blended finance tools to reduce interest rates, reduce risk to lenders, and increase the financial capacity of both lenders and borrowers to support broader health sector development without relying on donor funds. The use of credit guarantees and other blended finance tools may help mobilize funds from local financial institutions to help strengthen private sector service delivery by addressing the financing needs of private providers.

The need for social distancing during COVID-19 has also underscored the importance of harnessing digital technologies for HIV and other health services. The private sector has led innovation in telehealth, digital health information systems, and financial technologies. Many of these technologies can be used to increase the quality, efficiency, and effectiveness of HIV programs. Innovators increasingly deploy technological solutions to address long-standing public health challenges, coupled with financially viable business models and early-stage capital. Several locally owned and led companies use information technology and data systems as the backbone to transform pharmacies as the “community front door” for patients to access comprehensive primary care. In the next phase of the HIV response, there is an opportunity to ensure HIV services are available at pharmacies, affordable, and enabled by efficient digital technologies.

Greater government and donor engagement with the private sector may also ensure that innovative tools and technologies are developed and adopted strategically to increase interoperability with national electronic medical records systems and public health surveillance platforms. Interoperability supported by common data architecture and standards is a key opportunity where the private sector may be able to support national pandemic preparedness and response systems.

## Discussion

The aim of this paper is to inform the next generation of investments in private sector engagement within the global HIV response. This paper highlights program examples from USAID’s experience implementing activities under the PEPFAR-funded SFI. Other examples of private sector engagement activities exist beyond those mentioned here, both funded by PEPFAR and other sources. In the future, additional comparative analyses of these different approaches and their success factors would likely generate new insights and data to inform future investments. Further, regional, cross-sectional analyses may generate new lessons and insights based on the variation in private sector engagement activities across geographic regions and in different epidemiological contexts. Finally, additional research is needed to address the paucity of high quality, uniform, and systematically available data on private health sector access, utilization, and financing, including routine data on the number and type of private providers operating in a given area, the average cost of services, the uptake of services, and upstream market data on wholesale pricing and distribution options. Addressing these data gaps may allow host governments and donors to accelerate private sector engagement activities and more rapidly address issues of health equity.

## Conclusions

SFI’s activities over a multi-year period demonstrate the potential impact that engaging the private sector has on achieving and sustaining HIV epidemic control. Service delivery activities that trained private providers, increased their access to affordable HIV commodities, and improved logistics and information systems succeeded in increasing access to affordable, convenient HIV care for clients in the private sector. Financing activities decreased donor dependence through increasing private providers’ access to private loans and revenue generation opportunities. These approaches can be replicated in other LMICs to increase client-centered care and improve the financial sustainability of the HIV response. In the future, greater strategic planning and engagement between donors, governments, and the private sector can ensure that the private sector’s capabilities are harnessed to meet the 95-95−95 goals and control the HIV epidemic.

## Supplementary Information

Additional file 1. Program Approach and results of private sector engagement under SFI in 11 countries and two regional programs [40].

## Data Availability

The datasets used and/or analyzed during the current study are available from the corresponding author on reasonable request.

## Abbreviations

ART: Antiretroviral therapy
ARV: Antiretroviral medication
CBO: Community-based organization
CCMDD: Central Chronic Medicine Dispensing and Distribution
CSO: Civil society organization
DCA: Development credit authority
DR: Dominican Republic
EQUIP: Extending Quality Improvement Practices in Africa
EpiC: Meeting Targets and Maintaining Epidemic Control
GHSC-PSM: Global Health Supply Chain-Procurement and Supply Management
HIVST: HIV self-testing
HP+: Health Policy Plus
LMIC: Low-and middle-income country
NGO: Non-governmental organization
PEPFAR: President’s Emergency Plan for AIDS Relief
PLHIV: People living with HIV/AIDS
PMTCT: Prevention of mother to child transmission
PrEP: Pre-exposure prophylaxis
PSE: Private sector engagement
ROI: Return on investment
SFI: Sustainable Financing Initiative
SHOPS+: Strengthening Health Outcomes in the Private Sector Plus
SIDHAS: Strengthening Integrated Delivery of HIV/AIDS Services
USAID: U.S. Agency for International Development

## References

[1] UNAIDS. UNAIDS report on the global AIDS epidemic shows that 2020 targets will not be met because of deeply unequal success; COVID-19 risks blowing HIV progress way off course. Geneva: UNAIDS. 2020. https://www.unaids.org/en/resources/presscentre/pressreleaseandstatementarchive/2020/july/20200706_global-aids-report. Accessed 25 Oct 2021.

[2] Shubber Z, Mills EJ, Nachega JB, Vreeman R, Freitas M, Bock P et al. Patient-reported barriers to adherence to antiretroviral therapy: a systematic review and meta-analysis. PLoS Med. 2016. https://journals.plos.org/plosmedicine/article?id=10.1371/journal.pmed.1002183. Accessed 24 June 2021. 10.1371/journal.pmed.1002183 PMC5127502 27898679

[3] Kaye AD, Okeagu CN, Pham AD, Silva RA, Hurley JJ, Aaron BL et al. Economic impact of COVID-19 pandemic on healthcare facilities and systems: International perspectives. Best Pract Res Clin Anaesthesiol. 2020. https://www.ncbi.nlm.nih.gov/pmc/articles/PMC7670225/. Accessed 24 June 2021. 10.1016/j.bpa.2020.11.009 PMC7670225 34511220

[4] Waterfield KC, Shah GH, Etheredge GD, Ikhile O. Consequences of COVID-19 crisis for persons with HIV: the impact of social determinants of health. BMC Public Health. 2021;21(1):299. https://bmcpublichealth.biomedcentral.com/articles/10.1186/s12889-021-10296-9. Accessed 24 June 2021. 10.1186/s12889-021-10296-9 PMC7863613 33546659

[5] Grépin KA, Private Sector An Important But Not Dominant Provider Of Key Health Services In. Low- And Middle-Income Countries. Health Affairs (Millwood). 2016. https://www.healthaffairs.org/doi/10.1377/hlthaff.2015.0862?url_ver=Z39.88-2003&id=ori%3Arid%3Acrossref.org&dat=cr_pub++0pubmed. Accessed 25 June 2021. 10.1377/hlthaff.2015.0862 27385236

[6] Bollinger L, Bellows N. Examining potential private sector use for HIV services in six African countries [PowerPoint slides]. Efficiency and Accountability Technical Assistance Project II, Avenir Health. 2021. Accessed 21 July 2021.

[7] U.S. Department of State. About Us - PEPFAR [Internet], Washington DC. U.S. Department of State; [Cited 2021 Jun 24]. Available from: https://www.state.gov/about-us-pepfar/.

[8] UNAIDS. KFF/UNAIDS Analysis Finds Donor Governments Spent. US$7.8 Billion for HIV in 2019, Down Almost $200 Million From the Previous Year https://www.unaids.org/en/resources/presscentre/pressreleaseandstatementarchive/2020/july/20200706_kaiser-family-foundation . 2020. Accessed 24 June 2021.

[9] Sulzbach S, De S, Wang W. The private sector role in HIV/AIDS in the context of an expanded global response: expenditure trends in five sub-Saharan African countries, Health Policy and Planning. 2011;26(1);i72–i84. Available from: https://academic.oup.com/heapol/article/26/suppl_1/i72/558974. Accessed 25 June 2021. 10.1093/heapol/czr031 21729920

[10] Lee N, Cardenas M. The Future of Private Finance for Development in Poor Countries. Washington, DC: Center for Global Development. 2020. https://www.cgdev.org/publication/future-private-finance-development-poor-countries. Accessed 25 June 2021.

[11] Durosinmi-Etti O, Nwala EK, Oki F, Ikpeazu A, Godwin E, Umoh P et al. Willingness to Pay for HIV Prevention Commodities Among Key Population Groups in Nigeria. Global Health: Science and Practice. 2022. https://www.ghspjournal.org/content/10/5/e2100303#ref-1. Accessed 27 July 2023. 10.9745/GHSP-D-21-00303 PMC9622283 36316139

[12] Durosinmi-Etti O, Fried B, Dubé K, Sylvia S, Greene S, Ikpeazu A et al. Sustainability of Funding for HIV Treatment Services: A Cross-Sectional Survey of Patients’ Willingness to Pay for Treatment Services in Nigeria. Global Health Sci Pract. 2022;10(2):e2100550. https://www.ghspjournal.org/content/10/2/e2100550?utm_source=TrendMD&utm_medium=cpc&utm_campaign=Global_Health%253A_Science_and_Practice_TrendMD_0. Accessed 27 July 2023. 10.9745/GHSP-D-21-00550 PMC9053145 35487556

[13] Tran B, Nguyen L, Tran C, Le H, Tran S. The cost of antiretroviral treatment service for patients with HIV/AIDS in a central outpatient clinic in Vietnam. ClinicoEcon Outcomes Res. 2014;101. https://www.tandfonline.com/doi/full/10.2147/CEOR.S57028?scroll=top&needAccess=true&role=tab. Accessed 27 July 2023. 10.2147/CEOR.S57028 PMC3937113 24591843

[14] Tran BX, Fleming M, Nguyen CT, Latkin CA. Financial mobilization for antiretroviral therapy program: multi-level predictors of willingness to pay among patients with HIV/AIDS in Vietnam. AIDS Care. 2018;30(12):1488–97. https://www.tandfonline.com/doi/full/10.1080/09540121.2018.1503633?scroll=top&needAccess=true&role=tab. Accessed 27 July 2023. 10.1080/09540121.2018.1503633 30047280

[15] Thirumurthy H, Masters SH, Agot K. Willingness to Pay for HIV Self-Tests Among Women in Kenya: Implications for Subsidy and Pricing Policies. J Acquir Immune Defic Syndr. 2018;78(2):e8–11. https://journals.lww.com/jaids/Fulltext/2018/06010/Willingness_to_Pay_for_HIV_Self_Tests_Among_Women.18.aspx. Accessed 27 July 2023. 10.1097/QAI.0000000000001659 PMC5959049 29767641

[16] Johnson D, Cheng X. The role of private health providers in HIV testing: analysis of data from 18 countries. Int J Equity Health. 2014;13:36. https://equityhealthj.biomedcentral.com/articles/10.1186/1475-9276-13-36. Accessed 25 June 2021. 10.1186/1475-9276-13-36 PMC4054913 24884851

[17] McPake B, Hanson K. Managing the public–private mix to achieve universal health coverage. Lancet. 2016;388(10044):622–30. https://www.thelancet.com/journals/lancet/article/PIIS0140-6736(16)00344-5/fulltext. Accessed 27 July 2023. 10.1016/S0140-6736(16)00344-5 27358252

[18] Mackintosh M et al. What is the private sector? Understanding private provision in the health systems of low-income and middle-income countries. Lancet. 2016;388(10044):596–605. https://www.thelancet.com/journals/lancet/article/PIIS0140-6736(16)00342-1/fulltext. Accessed 27 July 2023. 10.1016/S0140-6736(16)00342-1 27358253

[19] Thurston S, Chakraborty NM, Hayes B, Mackay A, Moon P. Establishing and Scaling-Up clinical social franchise networks: lessons learned from Marie Stopes international and population services international. Global Health: Sci Pract. 2015;3(2):180–94. https://www.ghspjournal.org/content/3/2/180.abstract?utm_source=TrendMD&utm_medium=cpc&utm_campaign=Global_Health%253A_Science_and_Practice_TrendMD_0#sec-13. Accessed 27 July 2023. 10.9745/GHSP-D-15-00057 PMC4476858 26085017

[20] Corroon M, Kebede E, Spektor G, Speizer I. Key Role of Drug Shops and Pharmacies for Family Planning in Urban Nigeria and Kenya. Global Health Sci Pract. 2016;4(4):594–609. [cited 2021 Apr 9]. Available from: https://www.ghspjournal.org/content/4/4/594. Accessed 27 July 2023. 10.9745/GHSP-D-16-00197 PMC5199177 28031299

[21] Brugha R, Zwi A. Improving the quality of private sector delivery of public health services: challenges and strategies. Health Policy Plann. 1998;13(2):107–20. https://academic.oup.com/heapol/article/13/2/107/693569. Accessed 2023 July 27. 10.1093/heapol/13.2.107 10180399

[22] Baker S, Hijazi M, Nandakumar AK. Innovative approaches to HIV/AIDS financing: lessons learned from the Sustainable Financing Initiative (SFI). BMC Health Serv Res. 2025;24(Suppl 1). 10.1186/s12913-025-12529-8.10.1186/s12913-025-12529-840437408

[23] Chang J, Hijazi M, Baker S, Igboelina O, Mann C, Marqusee H, et al. Integrating HIV/AIDS services into financial protection systems to increase sustainability of the HIV/AIDS response. BMC Health Serv Res. 2025;24(Suppl 1). 10.1186/s12913-025-12528-9.10.1186/s12913-025-12528-940437441

[24] Mann C, Reuben E, Baker S, Hijazi M, Nandakumar AK, Shetty P et al. Addressing the HIV/AIDS investment gap through stronger public financial management system. BMC Health Serv Res. 2025;24(Suppl 1).10.1186/s12913-024-11324-1.10.1186/s12913-024-11324-140437478

[25] USAID. USAID Private Sector Engagement Policy, Washington DC, USAID. 2019. https://www.usaid.gov/sites/default/files/documents/1865/usaid_psepolicy_final.pdf. Accessed 25 Oct 2021.

[26] Chitah BM, Waters H. Assessment of the pilot decentralized drug delivery ART model and patient effectiveness. Zambia: [Unpublished] Lusaka; 2021.

[27] Office of the U.S, Global AIDS, Coordinator. Leveraging private pharmacists to expand ART distribution, promote adherence, and mobilize resources. Washington, DC: PEPFAR Solutions Platform. 2020. https://www.pepfarsolutions.org/solutions/2020/4/21/leveraging-private-pharmacists-to-expand-art-distribution-promote-adherence-and-mobilize-resources. Accessed 25 Oct 2021.

[28] Estevez I. Supporting access to finance for HIV service providers in Tanzania: Final activity report. Rockville, MD: Sustaining Health Outcomes through the Private Sector Plus Project, Abt Associates Inc. 2019. https://www.shopsplusproject.org/resource-center/supporting-access-finance-hiv-service-providers-tanzania. Accessed 25 Oct 2021.

[29] Sustaining Health Outcomes through the Private Sector Plus Project, Abt Associates Inc. Sustaining the HIV response in the Dominican Republic [video file]. 2021. https://www.shopsplusproject.org/resource-center/sustaining-hiv-response-dominican-republic. Accessed 2021 Oct 25.

[30] Mpofu M, Moyo T, Gilbert M, Dikobe W, Nishimoto L, Katiko G, et al. Distribution of antiretroviral therapy through private pharmacies and postal courier services during COVID-19 in Botswana: acceptability and reach of two out-of-facility individual differentiated service delivery models. J Int AIDS Soc. 2021;24:e25814. 10.1002/jia2.25814. Accessed 29 Oct 2021. 10.1002/jia2.25814 PMC8554222 34713589

[31] EpiC. Decentralized Drug Distribution in Cameroon: Final Report. Durham (NC): FHI 360. 2021. Available from: https://www.fhi360.org/wp-content/uploads/2024/02/epic-ddd-cameroon-english.pdf.

[32] FHI360. Meeting targets and maintaining epidemic control project. decentralized drug distribution country assessment results [PowerPoint slides]. 2020.

[33] Liu L, Christie S, Munsamy M, Roberts P, Pillay M, Shenoi SV et al. Expansion of a national differentiated service delivery model to support people living with HIV and other chronic conditions in South Africa: a descriptive analysis. BMC Health Serv Res. 2021; 21:463. 2021. https://bmchealthservres.biomedcentral.com/articles/10.1186/s12913-021-06450-z. Accessed 25 June 2021. 10.1186/s12913-021-06450-z PMC8127180 34001123

[34] Nichols BE, Cele R, Lekodeba N, Tukei B, Ngorima-Mabhena N, Tiam A et al. Economic evaluation of differentiated service delivery models for HIV treatment in Lesotho: costs to providers and patients. J. Int. AIDS Soc. 2021;24(4):e25692. https://www.ncbi.nlm.nih.gov/pmc/articles/PMC8035675/ Accessed 25 June 2021. 10.1002/jia2.25692 PMC8035675 33838012

[35] Nichols BE, Cele R, Jamieson L, Long LC, Siwale Z, Banda P et al. Community-based delivery of HIV treatment in Zambia: costs and outcomes. AIDS. 2021;35(2). https://www.ncbi.nlm.nih.gov/pmc/articles/PMC7810408/. Accessed 25 June 2021. 10.1097/QAD.0000000000002737 PMC7810408 33170578

[36] Dauda DS, Ugaz JI, Silfverberg D, Ogundipe A, Dutta A. Assessment of direct fees and indirect costs for people seeking HIV services in Nigeria. Washington, DC: Palladium, Health Policy Plus. 2019. http://www.healthpolicyplus.com/ns/pubs/17383-17697_NigeriaHIVUserFeeReport.pdf. Accessed 25 Oct 2021.

[37] Thurston S, Chakraborty NM, Hayes B, Mackay A, Moon P, Establishing and Scaling-Up Clinical Social Franchise Networks: Lessons Learned From Marie Stopes International and Population Services International. Global Health Sci Pract. 2015;3(2):180–94. https://www.ghspjournal.org/content/3/2/180.abstract?utm_source=TrendMD&utm_medium=cpc&utm_campaign=Global_Health%253A_Science_and_Practice_TrendMD_0#sec-13. Accessed 27 July 2023. 10.9745/GHSP-D-15-00057 PMC4476858 26085017

[38] Cashin C, Dossou JP. Can National Health Insurance Pave the Way to Universal Health Coverage in Sub-Saharan Africa? Health Systems & Reform. 2021;7(1). https://www.tandfonline.com/doi/full/10.1080/23288604.2021.2006122?scroll=top&needAccess=true&role=tab. Accessed 27 July 2023. 10.1080/23288604.2021.2006122 34965364

[39] Yu CP, Whynes DK, Sach TH. Equity in health care financing: The case of Malaysia. Int J Equity Health. 2008;7(1). https://equityhealthj.biomedcentral.com/articles/10.1186/1475-9276-7-15. Accessed 27 July 2023. 10.1186/1475-9276-7-15 PMC2467419 18541025

[40] MacDonald V, Callahan S, Basirika R, Ganesan R. Feasibility of Private Sector Delivery of Pre-Exposure Prophylaxis in Windhoek, Namibia. Rockville, MD: Sustaining Health Outcomes through the Private Sector Plus Project, Abt Associates Inc. 2020. https://shopsplusproject.org/resource-center/feasibility-private-sector-delivery-pre-exposure-prophylaxis-windhoek-namibia. Accessed 28 June 2021.

