# Supplementary material for: Engaging the private sector to increase access to HIV services and increase sustainability of the HIV response
Source: BMC Health Serv Res. 2025 May 28;24(Suppl 1):1672. doi: 10.1186/s12913-025-12530-1 (PMC12121042; doi:10.1186/s12913-025-12530-1)
Supplement: Supplementary file 1 — Additional file 1. Program Approach and results of private sector engagement under SFI in 11 countries and two regional programs [40]. [file 12913_2025_12530_MOESM1_ESM.docx]

**Engaging the private sector to increase access to HIV/AIDS services and increase sustainability of the HIV/AIDS response**

Additional file 1

Program approach and results of private sector engagement under SFI in 11 countries and two regional programs

| **Country** | **USAID- funded Partner** | **Activities** | **Results** | **Financial ROI (%)** |
| --- | --- | --- | --- | --- |
| Botswana | Meeting Targets and Maintaining Epidemic Control (EpiC) | Feasibility assessment and trial program of private pharmacy distribution and home delivery of ARVs, and viral load testing in private labs (2020-2021) | -Assessment indicated high interest and readiness among pharmacies to participate and high interest (61%) from clients [20].  -An exploratory trial of viral load testing in the private sector demonstrated feasibility, interest from clients and a short processing time. | N/A |
| Cambodia | Health Policy Plus (HP+) | Assessment of the potential of private for-profit sector contribution in HIV/AIDS in financing and service delivery (2019-2020) | Assessment found that the private sector is already actively involved in HIV service delivery. There is a further need for legal guidelines regulating the private sector and improved reporting. | N/A |
| Caribbean Region | Sustaining Health Outcomes in the Private Sector Plus (SHOPS+) | Feasibility study, design of operational framework and pilot of Caribbean tourism “Passport to Pride” fund to pool contributions from tourists to the Caribbean region into a fund that will provide grants to key population focused CSOs in the region (2017-2021) | Assessment indicated high potential for a fund to solicit donations from U.S.-based LGBT travelers and travel agents. A $137k donation for the fund’s startup was secured, the fund was designed, and marketing materials were developed. The launch of the fund was delayed due to COVID-19’s impact on the cruise and tourism sector and is anticipated to launch after the close of SFI. | -66% |
| Côte D'Ivoire | EpiC | Feasibility assessment of private pharmacy distribution of ARVs (2020-2021) | Assessment indicated high interest and readiness among pharmacies to participate and high acceptability (60%) from clients | N/A |
| Cameroon | EpiC | Evaluation of ART through community-based organizations and feasibility assessment of private pharmacy distribution of ARVs (2020-2021) | Findings indicated that CBO models of care resulted in superior retention in care, lower waiting time and higher patient satisfaction. Patients had a lower preference (12%) for pharmacy distribution compared to CBO (32%) or health facilities (49%). Private pharmacists were highly interested to participate. | N/A |
| Dominican Republic | SHOPS+ | 1) Introduce regulation to formalize private provider participation in national HIV care program (2020-2021)  2) Business development to improve the operational efficiency, implement social enterprise models and increase the financial sustainability of HIV-focused NGOs. Related activities that supported NGOs to contract with the national insurance program are described in *Integrating HIV/AIDS services into financial protection systems to increase sustainability of the HIV/AIDS response* (2018-2021) [23]. | 1) Drafted and validated a rule formalizing private providers to contract with the government for HIV services.  2) Provided business planning and capacity building support to 4 NGOs. As a result, one NGO received a grant and loan to launch a dermatology wing to cross-subsidize HIV services. | N/A |
| Ethiopia | HP+ | Assessment of willingness to pay and accept HIV services in private pharmacies and legal and regulatory assessment of the private HIV sector. (2020-2021) | Study protocols have been established but the assessment has been paused given the security situation in-country at the end of 2021. | N/A |
| India |  | Market assessment and design and piloting of an outcome-based strategic purchasing pilot for HIV testing and treatment services (2020-) | The assessment found that a main gap is initiation in treatment and retention at 12 months; the private sector has some key differentiators that demonstrate it can address these two gaps if given access to public sector supply chains. Pilot of an outcomes-based payment model in two districts is underway. |  |
| Kenya | 1) Global Health Supply Chain Program - Procurement and Supply Management (GHSC-PSM),  2 and 3)  HP+ | 1) Market assessment on the potential of increasing private participation in HIV treatment in Kenya (2018)  2) Finalization and adoption of a private sector engagement framework to allow for private sector uptake of ARVs (2018-2021)  3) Assessment of feasibility and willingness to pay for ART in private pharmacies (2020-2021) | 1) The assessment outlined a strategy for transitioning 80,000 HIV patients into the private sector and saving donors a projected $20 Million over 7 years.  2) Currently under development with NASCOP.  3) Mapping assessment prioritized private pharmacy ARV distribution in select counties. Client and pharmacy assessments indicated high interest in dispensing ARVs (95% of pharmacists) and moderate demand for private pharmacy pickup (46% of clients) at a maximum fee of 181-274 Kshs ($1.68 - 2.54 USD) per pickup. | N/A |
| Namibia | SHOPS+ | Assessment of demand and feasibility of private sector provision of PrEP (2018-2020) | Assessment indicated a potential market of 51,000 clients in the greater Windhoek metro area who could be served through PrEP distribution in the private sector [40]. | N/A |
| Nigeria | 1) Strengthening Integrated Delivery of HIV/AIDS Services (SIDHAS),  2) SHOPS+  3) GHSC-PSM | 1) Three private service delivery models were implemented in 4 States: a) a comprehensive treatment model in which clients received donor-funded ARVs and paid for clinical consultations and related services at private clinics, b) an ARV refill program in which public-sector clients picked up donor-funded ARVs at private pharmacies for a small dispensing fee, and c) partnerships with two private labs to provide viral load testing for clients willing and able to pay. (2016-2019)  2) Market assessment and strategy development of private HIV services in Lagos and Rivers states (2017-2018)  3) Assessment of the feasibility of commercial markets for ARVs, PrEP and HIV self test kits. Following the assessment, SFI negotiated with pharmacies and HIV self test kit distributors to increase affordability and access to HIV self testing in the private sector; and provided training to pharmacists on forecasting and supply management. (2018-2021) | 1) a) 4,484 clients accessed ART in private clinics; b) 15,266 clients accessed ART from private pharmacies; and c) 699 viral load tests were performed in the private sector. Private treatment models demonstrated superior retention outcomes, lower wait times and high patient satisfaction [27].  2) The assessment found that over 50% of Nigerians access health care from private providers, over 65% of providers in Lagos offer HIV testing and only 10% offer ART. The assessment offered a strategy for serving up to 30,000 more clients with HIV services in the private sector through market segmentation targeting undiagnosed and newly diagnosed higher income PLHIV.  3) The assessment found high demand for HIVST in the private sector and a viable market, but relatively lower feasibility for a commercial market for ARVs and PrEP. During implementation, 322 self test kits were purchased by private pharmacies to be retailed and 45 pharmacies were trained. | 1) 15% overall; a) -12%; b) 45%; c) -12%  2) -100%  3) N/A |
| Tanzania | SHOPS+ | 1) Pilot of 3 service delivery models: a)Support to Massana Hospital on business planning and pricing services; b) community-based ART with cost sharing (COCODA); c) integrated HIV care at nurse and midwife-led clinics (2017-2019)  2) Support formation of a partnership between the Trafigura Foundation and North Star Alliance to establish HIV services in roadside clinics catering to mining and transport workers (2017-2019)  3) A Development Credit Authority 50% credit guarantee along with training and mentorship to banks and providers encouraged CRDB bank to increase lending to private clinics (2017-2018)  4) Support expansion of Jamii, a mobile phone-based micro-insurance platform which enabled increase of private insurance coverage for HIV services (2017-2018)  5) Feasibility assessment and strategy development for the private distribution of HIVST (2017-2019) | 1) a) Costing revealed  that HIV services were  profitable and competitively priced;  b) 200 clients served with community-based ART for 2 pickups, after which pilot was put on hold. c) 7,222 clients tested and 240 initiated on ART  2) $1.4 million in grant funding leveraged to establish 6 roadside clinics at which 3,167 clients were tested and 101 clients were initiated on ART. 100% of those testing positive were linked to care.  3) 70 providers were coached on accessing loans and 26 loans totaling $6.4 million were disbursed to private providers, allowing them to purchase new equipment and expand their businesses. As a result, 17 providers increased HIV testing by 35%.  4)2,707 HIV clients were enrolled on insurance as a result of SFI efforts.  5) SFI analysis and advocacy contributed to a policy permitting use of HIVST, a first step towards private provision. | 1) a. N/A  b. -37%,  c. N/A  2) 775%  3) 1468%  4)-83%  5) N/A |
| Zambia | Extending Quality Improvement Practices in Africa (EQUIP) | Supported the scale up of a Central Dispensing Unit (CDU), which provided ARV packing, supply chain logistics, and distribution to a network of decentralized pickup points, including private pharmacies. (2019-2020) | 16,653 clients on ART | -39* |

*Zambia ROI is calculated including co-funding from USAID/Zambia
